# Real-World Experience With Brolucizumab 6 mg for Diabetic Macular Edema

**DOI:** 10.7759/cureus.52176

**Published:** 2024-01-12

**Authors:** Francisca Bragança, André Ferreira, João Leite, João Coelho, Tânia Borges, Filipa Caiado, Nuno Correia, João Beirão, Pedro Menéres, Bernardete Pessoa

**Affiliations:** 1 Ophthalmology, Centro Hospitalar Universitário de Santo António, Porto, PRT; 2 Unit of Anatomy, Department of Biomedicine, Faculty of Medicine, University of Porto, Porto, PRT; 3 Department of Ophthalmology, Instituto de Ciências Biomédicas Abel Salazar, University of Porto, Porto, PRT; 4 Unit for Multidisciplinary Research in Biomedicine, Instituto de Ciências Biomédicas Abel Salazar, University of Porto, Porto, PRT

**Keywords:** retina, diabetic retinopathy, diabetic macular edema, brolucizumab, anti-vascular endothelial growth factor

## Abstract

Background

This study aimed to assess functional and anatomical outcomes after one month of treatment with a single intravitreal injection (IVI) of brolucizumab in patients with diabetic macular edema (DME).

Methodology

A retrospective study was conducted on eyes with DME who received a single IVI of brolucizumab. The study was designed to assess visual function and optical coherence tomography (OCT) biomarkers at baseline and one month following a single brolucizumab IVI. A sub-analysis was conducted between the following two groups: group 1 - treatment with brolucizumab due to burden, needle phobia, or non-compliance (responders to standard anti-vascular endothelial growth factor (VEGF) or naïve); and group 2 - non-responsive to previous therapies (standard anti-VEGF ± corticosteroids). The main outcome measures included best-corrected visual acuity (BCVA; Early Treatment of Diabetic Retinopathy Study (ETDRS) letters), central foveal thickness, and OCT biomarkers such as the presence of subretinal fluid, the number of hyperreflective dots, the disorganization of retinal inner layers, the disruption of outer plexiform layer, external limiting membrane and ellipsoid zone, the presence of cysts in the nuclear layers (outer (ONL) and inner (INL)), and the number of cysts in ONL versus those in the INL. Safety outcomes were assessed.

Results

A total of 59 eyes from 42 patients were included, of which 47 eyes were in group 1 and 12 eyes were in group 2. At one month, patients had an improvement of two ETDRS letters on BCVA (p = 0.020), lower central foveal thickness (p < 0.001), fewer hyperreflective dots (p = 0.016), less outer plexiform layer disruption (p = 0.004), less inner and outer nuclear layer cysts (p < 0.001 and p = 0.001, respectively) and better relationship between ONL and INL cysts (p = 0.022). Results were significant in the subgroup of patients with previous responsive DME. No adverse events were reported.

Conclusions

This study demonstrates the effectiveness and safety after one injection of brolucizumab 6 mg in the management of DME, especially in previously responsive DME patients.

## Introduction

Diabetic macular edema (DME) has become the leading cause of diabetic retinopathy-related vision loss [1,2]. Anti-vascular endothelial growth factor (anti-VEGF) intravitreal treatment has become the first-line therapy for central DME [3]. In the retina, VEGF is a mediator in hypoxia-induced angiogenesis and increased vascular permeability [4,5]. Production of VEGF occurs through glial cells, such as Müller cells and astrocytes, as well as endothelial, vascular, and retinal pigmented epithelial cells [4]. In diabetic retinopathy, VEGF is produced by these cell types secondary to chronic hyperglycemia-induced biochemical pathways, such as diacylglycerol protein kinase C, advanced glycation end-products, hexosamine, and sorbitol pathways [4]. In addition to having direct effects on tight junction-associated proteins, VEGF interacts with oxidative stress mediators, pro-inflammatory cytokines, and chemokines produced in these pathways [6]. The endpoint of these reactions is vascular damage and blood-retinal barrier dysfunction, the hallmark of DME [6].

Brolucizumab is a humanized, monoclonal, single-chain, antibody fragment that selectively binds and inhibits major isoforms of VEGF-A. Due to its low molecular weight of 26 kDa, brolucizumab allows a molar dosing higher than aflibercept and ranibizumab [7]. Compared to anti-VEGF drugs, brolucizumab appears to bind with a higher affinity to VEGF-A [8]. High affinity, molar dosing, and small-sized molecules appear to impact brolucizumab’s retinal penetration and prolonged duration of treatment effect compared to the existing anti-VEGF drugs [8].

Approved since October 2019 in the United States for the treatment of neovascular age-related macular degeneration, brolucizumab 6 mg (BEOVU®, Novartis) has been approved by the European Medicines Agency, since March 28, 2022, and the US Food and Drug Administration, since June 3, 2022, for the treatment of visual impairment caused by DME. The approval followed 52-week results from two pivotal phase III, randomized, double-blinded global clinical trials (KITE and KESTREL) assessing the efficacy and safety of brolucizumab and aflibercept in DME patients. Data from these two trials showed the non-inferiority of brolucizumab in the primary outcome measure of best-corrected visual acuity (BCVA) change from baseline [9]. Concerning anatomical outcome measures, brolucizumab showed superiority in reducing central subfield thickness and subretinal and/or intraretinal fluid [9]. Overall, ocular and non-ocular adverse event rates were similar to those with aflibercept in each trial [9]. Subsequent results of these two pivotal trials at the 100-week mark showed that brolucizumab maintained BCVA gains and disease control, as measured by central subfield thickness improvement, compared to aflibercept, with fewer injections [10].

As treatment burden is an important risk factor for non-compliance in the management of diabetic patients [11], the increased durability of brolucizumab is an important feature to consider when planning a treatment regimen.

This study aimed to assess the functional and anatomical outcomes of a single intravitreal brolucizumab injection in patients with indication for treatment with brolucizumab due to burden, needle phobia, or non-compliance (responders to standard anti-VEGF or naïve) or those non-responsive to standard DME therapies (anti-VEGF ± corticosteroids).

## Materials and methods

A retrospective study was conducted among patients with DME treated with a single intravitreal injection (IVI) of brolucizumab 6 mg at the Ophthalmology Department of Centro Hospitalar Universitário de Santo António, Porto, Portugal, between May 2022 and September 2022. This study was designed to assess the functional efficacy of brolucizumab 6 mg through BCVA change from baseline to one month following treatment. Anatomical efficacy was assessed through quantitative and qualitative structural spectral-domain optical coherence tomography (SD-OCT) biomarkers at baseline and the one-month endpoint. A sub-analysis was conducted between the following two groups: group 1 - primary indication for brolucizumab due to treatment burden, needle phobia, or non-compliance (responders to standard anti-VEGF or naïve); and group 2 - non-responsive to previous therapies (standard anti-VEGF ± corticosteroids). The study was conducted according to the tenets of the Declaration of Helsinki in its latest amendment (Brazil, 2013) and was approved by the Ethics Committee of Health of Hospital de Santo António - Centro Hospitalar do Porto (HSA-CHP) (approval number: 2017.093 (084-DEFI/082-CES). All patients signed an informed consent form.

Study population

Eligible participants were patients with a diagnosis of type 1 or type 2 diabetes mellitus, who were over 18 years old and had an established diagnosis of central-involved DME, defined as central subfield foveal thickness >300 µm in the presence of intraretinal fluid on SD-OCT.

Eyes eligible for inclusion in group 1 exhibited SD-OCT characteristics of advanced DME [12] with a favorable response to standard anti-VEGF therapy but excessive treatment burden (a minimum of six IVIs every four to eight weeks) or inability to adhere to frequent anti-VEGF IVIs due to needle phobia or far distance from treatment zone. If indicated, both eyes of a patient could be included. Eyes of patients in group 2, the non-responsive DME group, were defined as having a reduction in central foveal thickness inferior to 20% after a loading dose of at least three standard anti-VEGF IVIs (aflibercept, ranibizumab, or bevacizumab) given at monthly intervals [13] or combined therapy with corticosteroid injections.

Exclusion criteria included patients with decreased visual acuity or macular edema that could be attributed to concomitant ocular disease that, except for the presence of cataract, included retinal vascular occlusion, clinically significant optic neuropathy, amblyopia, age-related macular degeneration, corneal opacity, macular hole, epiretinal membrane involving the fovea, significant vitreoretinal interface abnormality, and choroidal neovascularization. Patients with active or previous uveitis and active intraocular or periocular infection were excluded. Patients with any ocular surface/intraocular surgery (except cataract surgery and/or vitrectomy) and ocular trauma were also excluded. Patients who were unable to provide informed consent were excluded.

Study assessments

At baseline, patients were assessed for demographic data, diabetes mellitus type and duration, phakic/pseudophakic status, current eye topical medication, history of glaucoma or posterior segment surgery, laser therapy (macular laser and pan-retinal photocoagulation), and past intravitreal treatment (anti-VEGF and/or corticosteroids). Assessment of BCVA at baseline and one month after was performed using the Early Treatment of Diabetic Retinopathy Study (ETDRS) letters chart. The evaluation also included slit-lamp biomicroscopy, applanation tonometry, dilated fundus examination, and SD-OCT (Heidelberg Spectralis, Heidelberg, Germany).

SD-OCT parameters

Scans were obtained through SD-OCT (macular dense line scan mode HR 20 × 20° Spectralis HRA + OCT; Heidelberg Engineering, Heidelberg, Germany). This analysis was performed as described by Pessoa et al. [14]. In short, quantitative measurements were retrieved from the system and morphological parameters were assessed by two highly trained medical doctors (F.B. and A.F.), including the presence of subretinal fluid (SRF), the number of hyperreflective dots (HRDs), the disorganization of retinal inner layers (DRIL), the disruption of the outer plexiform layer (OPL), external limiting membrane (ELM) and ellipsoid zone (EZ), the presence of cysts in the nuclear layers (outer (ONL) and inner (INL)), and the number of cysts in the ONL versus those in the INL. DRIL was defined as a loss of definition of the boundaries of inner retinal layers. Disruption of OPL, ELM, and EZ was defined as an evident discontinuity in those layers. HRDs were defined as having a reflectivity similar to the nerve fiber layer and a dimension under 30 µ. The analysis of inner and outer nuclear layer cysts was done in light of the evidence that cysts located in the INL appear to be more responsive to anti-VEGF/corticosteroids than those in the ONL [15]. The presence or absence of cysts was documented, and the comparison between cysts in the ONL and INL was done subjectively by the investigators.

Statistical analysis

Statistical analysis was performed using SPSS version 29.0.0.0 (IBM Corp., Armonk, NY, USA). Descriptive statistics are shown as relative percentages of the total. The Kolmogorov-Smirnov test was used to assess the normal distribution of the data. Categorical variables were analyzed using chi-squared distribution and quantitative variables using the Mann-Whitney test. Statistical significance was defined as p-value <0.05.

## Results

A total of 59 eyes from 42 patients were included in this retrospective study. Of these, 47 were included in group 1, and 12 were included in group 2. Both naïve eyes of one patient were included in group 1 due to needle phobia.

Baseline characteristics

The mean age of the patients was 65.29 ± 12.4 years, with most being males (55.9%). Type 2 diabetes mellitus was the most prevalent type of diabetes (89.9%), with a mean duration of 18.12 ± 7.32 years. The majority of eyes (57.6%) were phakic and most (71.2%) had previous panretinal photocoagulation laser treatment. The median of previous anti-VEGF intravitreal injections before the switch was 11 (0-56). The mean baseline BCVA was 61.92 ± 17.63 ETDRS letters. Patients in group 1 were younger and had a shorter diabetes mellitus duration and better BCVA at baseline in comparison to non-responders (group 2), although these differences were non-significant. Patients in group 2 received more intravitreal corticosteroid injections (p = 0.007) in comparison with group 2. No other statistically significant differences were found between patient groups at baseline (Table 1).

**Table 1:** Baseline clinical and demographic characteristics of the study population. BCVA: best-corrected visual acuity; IV: intravitreal; PRP: panretinal photocoagulation; VEGF: vascular endothelial growth factor

Baseline characteristics	Overall	Group 1	Group 2	P-value
	(n = 59)	(n = 47)	(n = 12)	
Age (years), mean ± SD	65.29 ± 12.4	64.68 ± 12.68	67.67 ± 11.33	0.970
Female, n (%)	26 (44.1%)	20 (42.6%)	6 (50%)	0.643
Right eye, n (%)	32 (54.2%)	24 (51.1%)	8 (66.7%)	0.333
Type 2 diabetes mellitus, n (%)	53 (89.8%)	41 (87.2%)	12 (100%)	0.330
Diabetes mellitus duration, mean ± SD	18.12 ± 7.32	17.58 ± 7.38	19.83 ± 7.72	0.296
Phakic, n (%)	34 (57.6%)	27 (57.4%)	7 (58.3%)	0.956
BCVA, ETDRS letters, mean ± SD	61.92 ± 17.63	64.40 ± 15.14	59.58 ± 19.24	0.456
Glaucoma surgery, n (%)	6 (10.2%)	3 (6.4%)	3 (25%)	0.092
PRP, n (%)	42 (71.2%)	35 (76.1%)	7 (58.3%)	0.281
Macular laser therapy, n (%)	16 (27.1%)	11 (23.4%)	4 (33.3%)	0.480
Previous vitrectomy	14 (23.7%)	10 (21.3%)	4 (33.3%)	0.453
Cryotherapy, n (%)	2 (3.4%)	2 (4.3%)	0 (0%)	1.000
Anti-VEGF IV, median (range)	11 (0–56)	11 (0–56)	12.50 (3–53)	0.577
Steroid IV, median (range)	0 (0–9)	0 (0–4)	0.5 (0–9)	0.007

Brolucizumab 6 mg one-month response for the whole population and according to previous responsiveness status

At the one-month visit following brolucizumab 6 mg intravitreal administration, there was an overall statistically significant improvement in BCVA from baseline (p = 0.020) with a mean increase of 2.03 ± 6.29 EDTRS letters. Overall, there was a statistically significant improvement in central foveal thickness (CFT) (p < 0.001) and macular volume (MV) (p < 0.001). Regarding additional SD-OCT biomarkers, significantly lower HRD (p = 0.016), OPL disruption (p = 0.004), and INL (p < 0.001) and ONL cysts (p = 0.001) were verified at the one-month follow-up visit. There was also an improved relationship between ONL and INL cysts (p = 0.022). Other SD-OCT parameters such as SRF and ELM disruption also displayed improvement, although non-significant. In the sub-analysis, group 2 displayed no statistically significant improvement in clinical or SD-OCT biomarkers parameters from baseline. Meanwhile, in group 1, improvement in BCVA (p = 0.003), CFT (p < 0.001), MV (p < 0.001), OPL disruption (p = 0.006), INL cysts (p = 0.001), and ONL cysts (p < 0.001) was observed, along with a better relationship between ONL and INL cysts (p = 0.039). No systemic or ocular adverse events were reported during follow-up (Table 2).

**Table 2:** Brolucizumab 6 mg one-month response for the whole population and according to previous responsiveness status. BCVA: best-corrected visual acuity; CFT: central foveal thickness; DRIL: disorganization of retinal inner layers; ELM: external limiting membrane; EZ: ellipsoid zone; HRD: hyperreflective dots; INL: inner nuclear layer; IOP: intraocular pressure; ONL: outer nuclear layer; OPL: outer plexiform layer

Clinical and OCT-derived parameters	Total		Group 1		Group 2	
	(n = 59)	P-value	(n = 47)	P-value	(n = 12)	P-value
BCVA, mean ± SD						
Baseline	61.92 ± 17.63	0.020	62.51 ± 17.35	0.003	59.58 ± 19.24	0.889
1 month	63.95 ± 17.97		64.98 ± 17.60		59.92 ± 19.64	
CFT (µm), mean ± SD						
Baseline	381.97 ± 150.903	<0.001	340.96 ± 96.03	<0.001	454.83 ± 155.11	0.158
1 month	341.12 ± 130.930		311.69 ± 818.89		445.33 ± 218.29	
MV (mm^3^), mean ± SD						
Baseline	9.84 ± 2.04	<0.001	9.52 ± 1.50	<0.001	10.05 ± 2.29	0.182
1 month	9.35 ± 1.73		8.81 ± 1.29		9.99 ± 2.92	
Subretinal fluid, n (%)						
Baseline	8 (13.6%)	0.453	7 (14.9%)	0.375	1 (8.3%)	1.000
1 month	5 (8.5%)		4 (8.5%)		1 (8.3%)	
HRD, n (%)						
Baseline	21 (35.6%)	0.016	15 (31.9%)	0.063	6 (50%)	0.500
1 month	14 (23.7%)		10 (21.3%)		4 (33.3%)	
DRIL n (%)						
Baseline	1 (1.7%)	1.000	1 (2.1%)		0 (0%)	--
1 month	2 (3.4%)		2 (4.3%)	1.000	0 (0%)	
OPL disruption, n (%)						
Baseline	30 (50.8%)	0.004	22 (46.8%)	0.006	8 (66.7%)	0.625
1 month	18 (30.5%)		12 (25.5%)		6 (50%)	
ELM disruption, n (%)						
Baseline	12 (20.3%)	0.625	9 (19.1%)	0.500	3 (25%)	1.000
1 month	10 (16.9%)		7 (14.9%)		3 (25%)	
EZ disruption, n (%)						
baseline	12 (20.3%)	1.000	10 (21.3%)	0.500	2 (16.7%)	0.500
1 month	12 (20.3%)		8 (17%)		4 (33.3%)	
INL cysts, n (%)						
Baseline	31 (52.5%)	<0.001	25 (53.2%)	0.001	6 (50%)	0.250
1 month	17 (28.8%)		14 (29.8%)		3 (25%)	
ONL cysts, n (%)						
Baseline	47 (79.7%)	0.001	36 (76.6%)	<0.001	11 (91.7%)	1.000
1 month	33 (55.9%)		23 (48.9%)		10 (83.3%)	
ONL>INL cyst relation, n (%)						
Baseline	42 (71.2%)	0.022	31 (66%)	0.039	11 (91.7%)	1.000
1 month	33 (55.9%)		22 (46.8%)		11 (91.7%)	

## Discussion

This real-world study supports the effectiveness of brolucizumab 6 mg in the early treatment of DME only in patients previously responsive to anti-VEGF drugs. A single brolucizumab IVI was associated with a significant improvement in BCVA at the one-month follow-up visit, an improvement only significant in the group with naïve and previously responsive DME eyes. Regarding several SD-OCT parameters, although a significant improvement was observed for the pooled group, differences remained statistically significant only in group 1, namely, in lower CFT, OPL disruption, INL and ONL cysts, and an improved relation between ONL and INL cysts.

Treatment response in DME is an important topic that has been the subject of extensive investigation. The DCRC.net Protocols I and T found persistent macular edema to occur in approximately 40% of DME eyes after six monthly anti-VEGF IVIs [15-17]. The definition of poor response or unresponsiveness to anti-VEGF treatment in DME has been subject to discussion and there is no current consensus on a univocal definition [15]. Chronic persistent DME was defined by DRCR.net as eyes treated monthly with 0.5 mg ranibizumab that had not achieved a CST less than 250 µm and 10% or greater reduction relative to the six-month follow-up visit on at least two consecutive study visits [18].

In the early stages, DME appears to be mostly mediated by VEGF. The levels of this factor and other cytokines rise as DME or diabetic retinopathy progresses. Hypoxia primarily upregulates VEGF, with levels being especially elevated in cases of severe proliferative diabetic retinopathy [19-23]. Besides other systemic factors involved in DME pathogenesis [1,24,25], we can hypothesize that non-responsive DME may be caused by the unaddressed mediators or by the requirement for higher or more frequent doses of anti-VEGF/corticosteroids intravitreal therapy. In these non-responsive cases, the treatment burden becomes unacceptable and requires a proactive change in therapeutic strategy to achieve efficacy such as combining anti-VEGF with CCT, considering the delivery of 0.19 mg fluocinolone acetonide implant and/or pars plana vitrectomy [14,26]. An improved potency and longevity of brolucizumab’s action appear to be influenced by its smaller size, molar dosage, and stronger affinity to VEGF [7,8]. This is the rationale for using brolucizumab in DME cases unresponsive to anti-VEGF and/or corticosteroids or to reduce the burden related to standard anti-VEGF therapy.

Demographic and functional factors have been associated with a profile favoring anti-VEGF response, such as younger age and better baseline BCVA [15], which is in line with the differences found at baseline in our study, although non-significant. Biomarkers assessed through SD-OCT such as the presence of hyperreflective retinal spots, the disruption of ELM and EZ, and the disorganization of inner retinal layers have also been considered poor treatment response predictors [15]. In line with this evidence, our non-responder group exhibited higher percentages of HRD and ELM disruption at baseline and after treatment in comparison to previous responders. Vitreoretinal interface abnormalities such as epiretinal membrane, vitreomacular adhesion, and vitreomacular traction have also been associated with a poorer response, although this was not the focus of our study [27]. Hence, systemic and ocular factors can be used to help predict treatment response. As observed, previous non-responders to anti-VEGF are more likely to exhibit complex anatomical macular findings, and 1 brolucizumab IVI may be insufficient to address real outcomes in the long term.

To our knowledge, this is the first study reporting the effect of brolucizumab in OCT biomarkers proposed by the European School for Advanced Studies in Ophthalmology classification for grading of diabetic maculopathy [12].

Our study found that those who were responding favorably to other anti-VEGF intravitreal drugs, when injected during the window effect of previous therapies, continued to retrieve positive functional and anatomical outcomes after one injection of brolucizumab, with the additional potential benefit for a lower treatment burden through less frequent hospital visits and anxiety-associated procedures.

As the approval of brolucizumab in the treatment of DME is recent, real-world studies on brolucizumab’s effectiveness and safety are still scarce. Murray et al. [28] retrospectively reviewed intravitreal brolucizumab as a rescue therapy in patients with unresponsive macular edema due to diabetic retinopathy and other conditions such as radiation retinopathy and epiretinal membrane. All eyes included had been previously treated with a mean of 15 injections of intravitreal bevacizumab and/or intravitreal triamcinolone acetonide. A total of 13 eyes with complex, chronic DME showed statistically significant improvement in BCVA and CFT six weeks after brolucizumab administration, without major adverse events. Chakraborty et al. [29] reported a case of a 48-year-old male patient with bilateral DME who underwent brolucizumab IVI only in the left eye, which exhibited a CFT of 637 µm. Brolucizumab was not administered to the right eye due to financial constraints. At the one-month follow-up visit, an improvement in BCVA in the left eye from 20/120 to 20/60 was observed, alongside an improvement in CFT (248 μm). Interestingly, an improvement in BCVA and CFT was also documented in the right eye. The authors postulate this contralateral effect may be due to systemic escape of the molecule or due to the disease’s natural progression. A retrospective interventional single-center study was conducted by Chakraborty et al. [30] in 13 eyes of 13 patients with chronic, non-responsive, center-involved DME. Eyes had previously been treated with IVI of anti-VEGF alone or in combination with IV corticosteroids. Each patient, except one, received two brolucizumab IVIs, the second being administered when the visual acuity declined by one Snellen line or when the CFT increased by 30%. On the 12-week follow-up visit after the first injection, BCVA and CFT improved significantly. Among the patients who completed 16 weeks of follow-up after the first brolucizumab IVI, 91.6% could achieve 16 weeks of treatment-free interval. There were no reported cases of intraocular inflammation, occlusive vascular events, or retinal vasculitis in any of these real-world studies.

In KESTREL and KITE, at 52 and 100 weeks, adverse events considered of special interest included intraocular inflammation, including retinal vasculitis, retinal vascular occlusion, and endophthalmitis, which exhibited similar incidence between brolucizumab and aflibercept [9,10]. In our study, there were no systemic or ocular adverse events, although the brief follow-up period limits robust long-term conclusions on safety.

Our study has other limitations, including a relatively small sample size, as expected given it’s been only available recently, and the drawbacks inherent to the retrospective design, as well as a short follow-up. Yet, our study adds relevant real-world experience on this subject as this drug was recently approved in Europe and only a few studies have been conducted.

## Conclusions

This study aims to contribute to the growing knowledge of the effectiveness and safety of brolucizumab in real-world settings in the treatment of DME. For DME patients who failed anti-VEGF treatment, more research needs to be undertaken, as a single brolucizumab IVI is most likely insufficient to address the disease complexity of these cases. In previously responsive patients, brolucizumab conveyed functional and anatomical benefits with a single injection, while potentially reducing treatment burden and increasing compliance in the long term.
